# The Department of Modern Nursing

**Published:** 1915-04-03

**Authors:** 


### THE DEPARTMENT OF MODERN NURSING.*

### XI.—The Royal Infirmary of Edinburgh.

There could scarcely be a more striking example of the evolution of nursing, during half a century, than is to be found in the Royal Infirmary of Edinburgh. Less than fifty years ago, the legend runs, each doctor and surgeon regarded it as an important part of the daily routine to go and choose the most sober member of the nursing staff he could find to take charge of his ward in the night. So limited, indeed, were his resources, in the matter of securing anyone really competent, that, with a critical case, it was customary to establish a medical student as special nurse. Yet, out of the old infirmary in the city's slums, with dingy wards and staffed by Gamps of varying degrees of cleanliness and sobriety, there has developed one of the most magnificent training schools in the world. When one is conversant with the organisation and teaching of this great school of nursing, and compares its standard of nursing efficiency with that which would be set up by the proposed Nursing Council, the thought recurs with increasing insistency that the ideal of the advocates for Registration falls far short of the standard already established in the more highly developed nursing schools of the day.

The Royal Infirmary has the reputation of being a very hard training school. Those entering as probationers are soon alive to the fact that, before its valuable certificate will be placed in their hands, much will be required of them. Yet, to the enthusiastic nurse, this proves no discouragement; on the contrary, the whole system of the school tends to develop her best energies and aspirations with a certain sub-conscious knowledge, too, that, even to the performance of the most trivial duties allotted her, nothing but her best will prove good enough for the hospital. A quickly developed mental habit of regarding her work as of the first importance in her hospital life is fostered by the rigid discipline maintained, and has no small share in developing thorough efficiency and ensuring a successful career when the term of training is ended.

The Nurses' Home, with its pretty gables and walls overrun by creepers, presents rather a pleasing contrast to the stately pavilions of the hospital itself. In all its appointments the home is well calculated to provide a maximum of comfort for the nursing staff. Bedrooms are simply but comfortably furnished, and, in them, adequate provision is made for ventilation, lighting, and heating. The rooms of the night nurses are so apportioned as to ensure absolute quiet in the daytime. The recreation room in the home is a beautiful and spacious apartment, luxuriously furnished, in which nurses may, when off duty, rest and amuse themselves, with the one restriction that, during the hours when the night nurses are asleep, the piano is kept closed. In the library, which is situated in another part of the hospital, the strictest quiet is enforced. It is suitably furnished and supplied with all the important periodicals of the day, while its well-stocked bookcases afford ample opportunity for study. The nurses' sickrooms are bright, pleasant rooms in a quiet part of the home. Kitchen offices and sisters' duty room are attached. The enormous dining room, the home kitchen, and the office of the home superintendent are situated in a part of the hospital building, and these are connected with the home by a fine conservatory which, on one side, opens into the garden adjoining the nurses' home and, on the other, to the nurses' tennis courts. The home itself is built round a cool quiet court, a favourite spot in summer-time, with a fountain playing in its centre. The nurses are all extremely well satisfied with their home, the one complaint to be heard being that there is so little time to spend in it.

Strict vigilance is maintained as regards the hours for sleep, and provision is made to ensure that all lights are extinguished, with the exception of those which may be necessary in the sickroom, by 10.30 p.m. At a quarter to six in the morning a bell is rung throughout the corridors in the home, and, before going on duty in their wards at seven, probationers, as the first-year nurses are termed, must attend prayers, appear at the breakfast table, and answer to the roll-call. At 8.30 p.m. they leave their wards, and, after supper and prayers, are free until 10 p.m. to spend their time in the home or garden as they please. Obviously, during the day there must be relays of nurses for each meal. At 9.30 to 10 a.m. they take it in turn to go to a meal, usually spoken of as lunch, which consists of fish, meat, and sweets. Dinner is served to the day nurses at 2.30 and 3 p.m., and at each table a superintendent presides. This arrangement might well be imitated by many superintendents and matrons as tending to encourage good catering and cooking, and to ensure that meals are served in a proper manner. Tea is really the only meal at which no superintendent is present. The night staff dines at 8.30 a.m.—half an hour after coming off duty. It is a debateable point whether this is the most satisfactory and suitable arrangement from the nurses' point of view. Although it is a general practice, the fact that many of the most unpleasant duties in the ward routine fall to be performed during their last two hours on duty is not calculated to act as a stimulus to appetite. Added to this, the nurses are tired and apt to be less inclined to eat than when they come fresh from the day's rest.

It would be interesting to try the effect of a reversal of the present plan which provides a substantial meal, corresponding to the ordinary breakfast, half an hour before going on duty, and dinner half an hour after coming off duty—*i.e.*, to serve dinner before coming on duty, and a light meal on leaving duty.

* Previous articles: May 16, 30; June 13; July 11, 25; Aug. 8, 1914; Jan. 16, 30; Feb. 27; March 13, 1915.

Blair House, a beautiful little Home of Rest for the nurses, lies close to the hills at Colinton. The nurses are sent here to recruit after illness or to get any rest, additional to ordinary holidays, of which the lady superintendent or medical man may consider that they stand in need. On the whole, the health of the nursing staff is exceptionally good.

The average number of nurses employed in the hospital is some 280, and this number will shortly be increased. It does not include those working in the large Convalescent Home for patients at Murrayfield, and time spent there is not counted as training. The average number of patients in the hospital is 856, and the beds and cots number 961 (*i.e.* 921 beds and 40 cots). There are thirty-two sisters and some twenty-three fully certificated nurses, besides day nurses at various stages of their training. It is not until a nurse has been some eighteen months at least in the hospital that her services are regarded as being of any real value to it. From seventy to seventy-five probationers enter for training during the year, and some nine or ten out of this number leave from various causes at the end of their third month. Before entering hospital, candidates for training must first pass an examination which, however, is merely a means of ascertaining whether their general education has been such as to ensure their being able to take an intelligent grasp of what is taught them.

Practical training in the wards is most comprehensive and thorough. The medical and gynæcological wards (two) each contain some thirty beds or more, and consequently afford wide opportunities for gaining experience. There are forty-eight beds in the eye wards, twenty in the skin wards, twenty-two in the ear and throat wards, and twenty-three in the mental and delirous, etc. ward. On the surgical side of the hospital the wards are smaller, but there each sister is responsible for double wards—one male and one female. Nurses in training work first in one and then in the other, and are present at a certain proportion of the operations. The nurses have the opportunity of learning theatre-work, sterilising, and preparation of dressings. From time to time the question is raised whether probationers should or should not be required to dust their wards, polish brass, care for the plants, and do similar domestic duties. Within reasonable limits, however, this kind of work is of real importance, for a knowledge of how these duties should be performed not only tends to make the nurses take more pride in the appearance of their wards, but it also leads to a clearer knowledge of exactly when and where those working under them fall short.

Though they have little actual responsibility, probationers are taught all the treatment carried out in the wards; they are made conversant with the reasons why it is prescribed and are expected to take an intelligent interest in its results. It is the duty of the sisters and the senior nurses to take every possible opportunity for teaching those less advanced, and the strictest supervision is maintained over the teaching of nurses in training. Some of the sisters are admittedly better teachers than others, and it might add somewhat to the thoroughness of the practical teaching if each sister were made personally responsible for her probationers becoming proficient before leaving her ward in certain definite lists of duties in which the particular ward offers opportunities for training. The onus of profiting by her training, however, must in the end rest with the probationer herself, and it is common knowledge that, while some nurses make the most of their opportunities, others have a wonderful ability for merely marking time.

There are arrangements for securing for every nurse opportunity for practical training in all the special wards, and so, in comparison with other hospitals, the training given provides great variety.

Courses of lectures are given to the nurses by members of the honorary medical staff on medical nursing, surgical nursing, anatomy, physiology, hygiene, gynæcology, and *materia medica*, and also two lectures are given every year on each of the following subjects—eye, ear, nose, throat, and skin treatment. Classes are also conducted by one of the house surgeons on bandaging and instruments. In addition to, and usually in connection with, those lectures and classes, bandaging and study classes are held by the assistant superintendents, while classes on sickroom cookery are undertaken by the kitchen mistress and classes on pharmacy by the chief dispenser. On all those subjects separate examinations are held, and prizes are awarded to those proving themselves to be most efficient. Advanced lectures on various subjects are also given to the senior nurses, and other nurses, not connected with the hospital but working in the city, are allowed to attend them.

At the end of her first year, which is spent on day duty, each nurse, for a period of six months, acts as assistant night nurse and then is again on day duty for six months. After this she is usually put in charge of a ward during the night for six months. This is regarded as a very valuable part of her training, as she has greater and more educative responsibility than during any previous period. On coming off night duty for the second time the nurse acts as staff nurse or as assistant nurse in different wards, and, in her fourth year, she may be appointed to take charge of the ward in the absence of the sister on holiday, and she also does three months' night duty.

The amount of off-duty time allowed varies somewhat according to the probationer's status. Roughly speaking, nurses have two hours off duty daily, one half-day in one fortnight, and a whole one in the next, while each nurse is allowed to go off duty for one service on Sunday, taking morning, afternoon, or evening in rotation. Night nurses are off duty for two hours and a half daily, and for one night each month. The length of holidays allowed depends upon the period of training at which the nurse has arrived. Probationers have three weeks' holiday in the year, and other nurses have a month, while the sisters have five weeks.

In an enormous institution nurses can have little opportunity for experience in ordinary domestic management. The sister requisitions the diets for patients from the hospital kitchen or stewards' department, as the case may be. The staff nurse usually has charge of the ward linen, although the sister is held directly responsible for its condition.

There is no private staff connected with the hospital, and the salaries paid to the nurses vary. Probationers receive £8 annually, second-year nurses £14, third-year nurses £18, and fourth-year nurses £25 annually. If a nurse remains for a fifth year the salary is £30. There is a pension scheme for the sisters. Each nurse may be said to cost the hospital some £170 to £180 at least during her period of four years' training.

During 1913 three of the trained nurses were made sisters in other hospitals, two were appointed under the Colonial Nursing Service, one was appointed to Queen Alexandra's Imperial Nursing Service, two joined the Queen Victoria Jubilee Nursing Institute, two went abroad, two to mission work, two entered for fever training and four for midwifery, twenty-six became private nurses, eleven went into nursing homes, one was married, while four went to their homes. Twenty probationers left, eight of those having proved unsuitable; three went on account of their health, six by their own wish, two married, and one died.

